# Measurement‐based study on characterizing symmetric and asymmetric respiratory motion interplay effect on target dose distribution in the proton pencil beam scanning

**DOI:** 10.1002/acm2.12846

**Published:** 2020-03-14

**Authors:** Eunsin Lee, Daniel Perry, Joseph Speth, Yongbin Zhang, Zhiyan Xiao, Anthony Mascia

**Affiliations:** ^1^ Department of Radiation Oncology University of Cincinnati College of Medicine Cincinnati OH USA; ^2^ Cincinnati Children’s Hospital Medical Center Cincinnati OH USA; ^3^ Department of Radiation Oncology University of Cincinnati Medical Center Cincinnati OH USA

**Keywords:** conformity index, homogeneity index, interplay effect, motion platform, proton pencil beam scanning, respiratory motion

## Abstract

Pencil beam scanning proton therapy makes possible intensity modulation, resulting in improved target dose conformity and organ‐at‐risk (OAR) dose sparing. This benefit, however, results in increased sensitivity to certain clinical and beam delivery parameters, such as respiratory motion. These effects can cause plan degeneration, which could lead to decreased tumor dose or increased OAR dose. This study evaluated the measurements of proton pencil beam scanning delivery made with a 2D ion chamber array in solid water on a 1D motion platform, where respiratory motion was simulated using sine and cosine^4^ waves representing sinusoidal symmetric and realistic asymmetric breathing motions, respectively. Motion amplitudes were 0.5 cm and 1 cm corresponding to 1 cm and 2 cm of maximum respiratory excursions, respectively, with 5 sec fixed breathing cycle. The treatment plans were created to mimic spherical targets of 3 cm or 10 cm diameter located at 5 cm or 1 cm depth in solid water phantom. A reference RBE dose of 200 cGy per fraction was delivered in 1, 5, 10, and 15 fractions for each dataset. We evaluated dose conformity and uniformity at the center plane of targets by using the Conformation Number and the Homogeneity Index, respectively. Results indicated that dose conformity as well as homogeneity was more affected by motion for smaller targets. Dose conformity was better achieved for symmetric breathing patterns than asymmetric breathing patterns regardless of the number of fractions. The presence of a range shifter with shallow targets reduced the motion effect by improving dose homogeneity. While motion effects are known to be averaged out over the course of multifractional treatments, this might not be true for proton pencil beam scanning under asymmetrical breathing pattern.

## 1 Introduction

Highly conformal proton pencil beam scanning (PBS) dose distributions generated with intensity‐modulated proton therapy (IMPT) improve the therapeutic ratio, achieving highly conformal target doses while reducing toxic doses to surrounding organs‐at‐risk (OARs). However, since there is interference between PBS delivery and moving target, known as the interplay effect,^1^, ^2^ and typical breathing cycles are on the same scale as the time required for the switch between two adjacent energy layers,^3^ the superior dose distribution is more sensitive to respiration‐induced organ motion for treatment sites such as lung, liver, and mediastinum, which can cause temporal displacement of the target volume and thus degrade the proton dose distribution significantly.^4^, ^5^ It was shown that during dynamic proton beam scanning, intrafractional organ motion induces up to 100% of the target to receive a dose outside the International Commission on Radiation Units and Measurements (ICRU) recommended limits with a minimal dose down to 34% of the prescribed dose in the extreme cases.^6^ To mitigate the motion interplay effect, several methods including respiratory gating, breath hold, tumor tracking, and repainting have been investigated or clinically implemented.^7^, ^8^, ^9^, ^10^, ^11^, ^12^

There were several studies on interplay effects for dynamic delivery of charged particles. Fundamental water phantom‐based computer simulation study of dose distribution in the presence of respiratory motion with extensive parameters was performed in the Paul Scherrer Institute,^13^ which showed rescanning the target volume with fractionation improves the dose uniformity. Homogeneity degradation of dose distribution with increasing motion of moving target was shown using radiographic film measurements and confirmed by real patient 4DCT‐based treatment planning study.^1^ Further studies including motion alleviation techniques such as increasing number of fractions or number of scanning of the target in order to mitigate interplay effects were investigated. The different repainting techniques^3^ as well as different scanning modes^2^, ^14^ in PBS were also investigated. Gating and rescanning combined phase‐controlled rescanning has also been studied for carbon spot scanning.^15^ Boria et al^16^ investigated the interplay effect mitigation of PBS in terms of fractionation on real pediatric patient 4DCT dataset.

Recently, Pfeiler et al^17^ has shown the implementation of a 4D dose calculation routine for PBS using the time structure of the pencil beam spot delivery from a system log files with validation via 2D array ion chamber and motion platform. Fraction‐wise retrospective dose reconstruction and accumulation was investigated using machine log files in combination with the patient’s breathing patterns from a pressure belt system and 4D CT datasets through entire treatment course.^18^ The deforming grid 4D dose calculation techniques have been employed to predict and validate the pattern of 4D dose distribution^19^ and to evaluate different PBS rescanning techniques for moving targets.^20^ Several studies have investigated 4D robust optimization for mitigating the interplay effects in scanned particle beam therapy. 21, 22, 23, 24

Whereas most of the studies were performed using simulation models in planning data or in phantom model, our investigation is solely measurement‐based study by delivering PBS plans, where the actual fractional dose of 200 cGy was delivered multiple times for a given number of fractions. The goal of this study is to investigate the interplay effect of PBS for different breathing patterns: sinusoidal symmetric motion vs more realistic asymmetric motion, which are simulated by a commercially available respiratory motion platform. The dosimetric influence under different sizes of targets and motion amplitudes with different spot sizes are evaluated using a conformity index and a homogeneity index and as a function of fractionations. This quantifies and demonstrates how different fractionation mitigates interplay effect with different breathing patterns based on real measurements of the delivery of PBS plans.

## 2 Materials and Methods

### 2.A Pencil beam spot scanning delivery system

Varian ProBeam^®^ system (Varian Medical Systems, Palo Alto, CA) uses active dynamic pencil beam scanning that can deliver IMPT with average dose rate of 2 Gy/L/min across the energy range of 70–244 MeV. The average time per energy layer switch is less than 1 sec and the minimum time to deliver the minimum weighted spot per energy layer is ~ 3 ms. A typical nozzle current is ~ 2 nA during patient treatment. Spot size measured in air has a sigma of 3.8 mm to 5.6 mm corresponding to 244 to 70 MeV at isocenter. With 5 cm of water equivalent thickness (WET) range shifter, each spot size increases roughly three times larger than one without range shifter. The deliverable minimum monitor unit (MU) per spot is 2 MU.

### 2.B Treatment plans

Treatment plans were generated in Eclipse^TM^ treatment planning system (Varian Medical Systems, Palo Alto, CA) for simulating small (3 cm diameter sphere) or large (10 cm diameter sphere) targets situated at 1 cm and 5 cm depths, where depth is defined as the distance from the water surface to the proximal surface of a sphere. Shallow targets at 1 cm depth were designed for benchmarking motion impact from larger beam spot sizes, where 5 cm of WET range shifter had to be inserted in the nozzle due to the cyclotron’s lowest energy limit (70 MeV corresponding to range of 4.1 cm). All target volumes were well covered by 95% isodose line, where reference RBE dose of 200 cGy per fraction was delivered at 1, 5, 10, and 15 fractions for each dataset.

For small target of 3 cm diameter sphere at shallow depth (with RS), 94.5–115.5 MeV energy spectrum of eight layers with 119 spots and at deeper depth (no RS), 79.5–103.5 MeV, nine layers, 296 spots were used. For large target of 10 cm diameter sphere at shallow depth (with RS), 93.6–156.6 MeV energy spectrum of 22 layers with 1488 spots and at deeper depth (no RS), 81.3–147.3 MeV, 23 layers, 4615 spots were used. Delivery time of a single fraction of 200 cGy for the small target is about 15 sec and 65 sec for the large target.

### 2.C Respiratory motion simulation and measurements

**FIG. 1:**
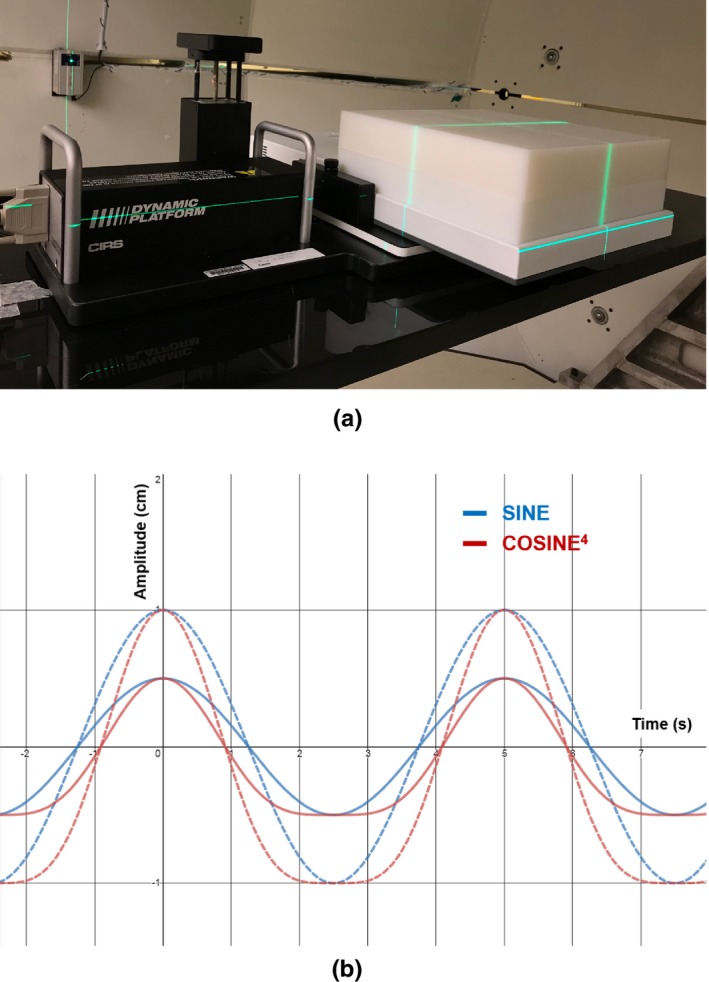
Measurement setup with 2D ion chamber array detector placed on top of respiratory motion platform (a). Sinusoidal symmetric and more realistic asymmetric breathing patterns generated from the motion platform (b). For a fixed breathing cycle of 5 sec, sine (sinusoidal symmetric) and cosine^4^ (realistic asymmetric) motions are generated with amplitudes of 0.5 cm and 1 cm.

The Dynamic Platform Model 008PL (CIRS, Norfolk, VA) was used for simulating respiratory motion as shown in [Fig. 1(a)]. The motion data were acquired in three different 1D motion ranges (±0.5 cm, ±1.0 cm, and ± 2.0 cm) with a fixed breathing cycle of 5 sec. For symmetric breathing pattern, a sinusoidal sine function was generated whereas for realistic asymmetric breathing pattern, a cosine^4^ function was used, which consists of inspiration of 1.8 sec and expiration of 3.2 sec, spending 64% of breathing period for expiration. All the measurements were made with 2D ion chamber array detector, MatriXX (IBA Dosimetry, Bartlett, TN) laid on the dynamic motion platform. Depths were simulated with solid water phantoms placed on the MatriXX detector. The measurement setup is shown in [Fig. 1(a)]. For each motion parameter set, a single fractionated plan was delivered 15 times independently and each of the single fractionated delivery was measured separately. In other words, fractionation was measured using a random initial phase for each fraction. Then, the number of single fraction measurements was added up accordingly to make a multifraction measurement data.

### 2.D Evaluation metrics and analysis

In order to provide a dose conformity score, instead of using a conformity index defined as the ratio of reference isodose volume to target volume, which is described in the ICRU Report 62^25^ we used a metric called conformation number (CN) introduced by van’t Riet et al^26^:

(1) $$CN=\frac{TV_{\mathrm{RI}}}{\mathrm{TV}}\times \frac{TV_{\mathrm{RI}}}{V_{\mathrm{RI}}}$$

where $TV_{\mathrm{RI}}$ is target volume covered by the reference isodose, TV is target volume, and $V_{\mathrm{RI}}$ is volume of the reference isodose. In this study, 95% of the prescribed dose is used as the reference isodose. The first term in the right‐hand side of Eq. (1) is a modified conformity index that correctly determines the quality of irradiation of the target volume, where 0 and 1 indicate that none of the target volume is located inside the prescription isodose and entire target volume is covered with the prescribed dose, respectively. The second term measures indirectly the volume of surrounding normal tissues involved in the reference isodose in terms of the degree of concordance between target volume covered with the reference isodose and the reference isodose volume, ranging from 0 (no protection of OARs) to 1 (all OARs below the reference isodose). For this study relative CN was used, which was normalized to the CN with no motion since the coverage or conformity for each plan depends on its margin, which is not identical for each different target size and depth.

Homogeneity index ( HI ) was also used to analyze the uniformity of dose distribution in the target volume. Among various formulae, we chose one defined as follows^27^

(2) $$HI=\frac{D_{2\%}-D_{98\%}}{D_{\mathrm{Rx}}}$$

where $D_{\mathrm{Rx}}$ is prescribed dose and $D_{2\%}$ and $D_{98\%}$ are the minimum doses to the 2% and 98% of the target volume, respectively. These are also considered to be maximum and minimum dose, respectively. In this study, we used $D_{\max}$ for $D_{2\%}$ and $D_{\min}$ for $D_{98\%}$ , which are practically equivalent considering 2D array ion chamber detector resolution (~0.78 cm) and the number of voxels covered by targets. Lower HI values indicate more homogeneous target dose distribution.

## 3 Results

**FIG. 2:**
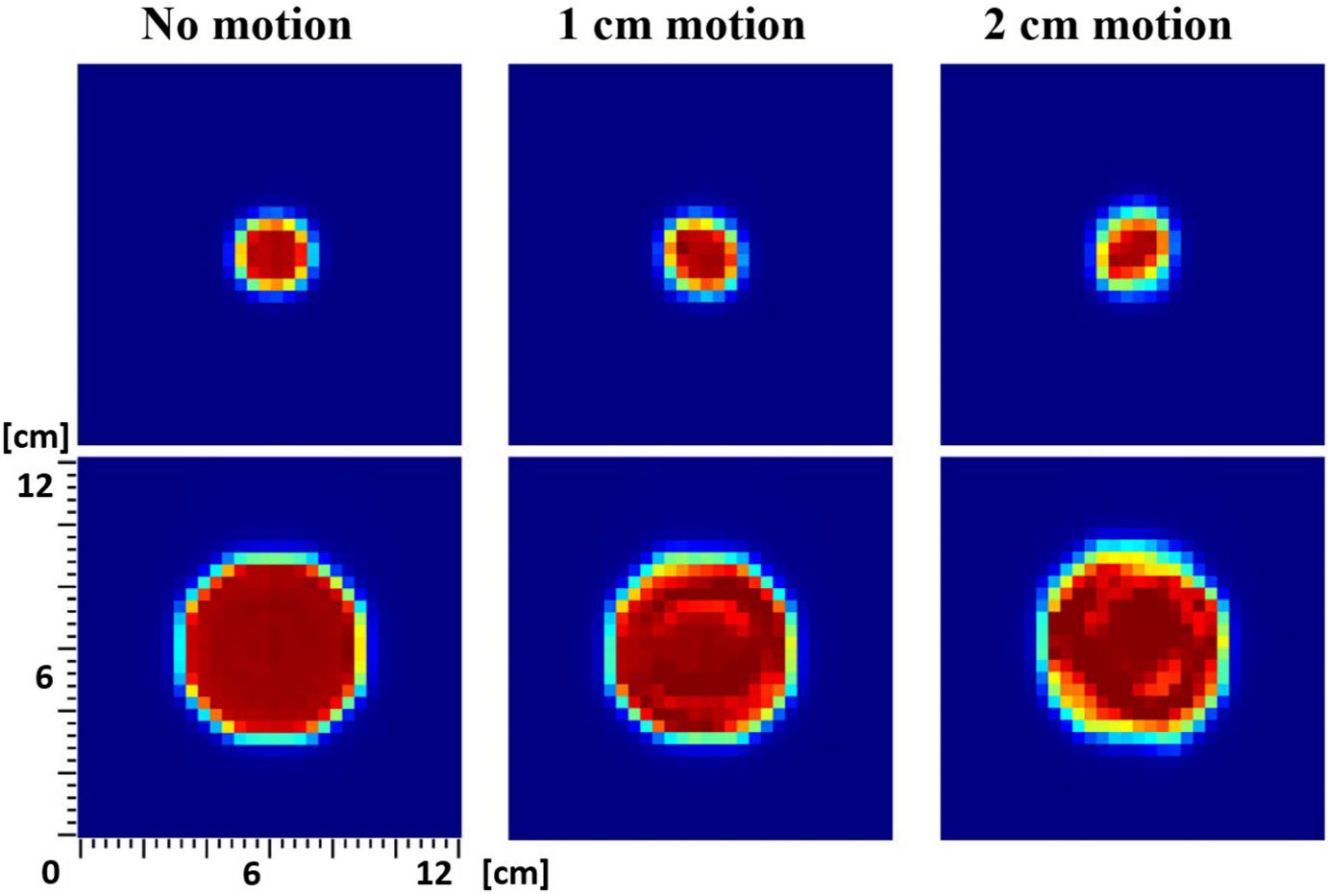
Two‐dimensional array ion chamber measurements of single fraction dose distribution with a fixed 5‐second breathing cycle for targets of 3 cm (top) and 10 cm (bottom) diameter spheres at 5 cm depth for asymmetric (cosine^4^) breathing pattern as a function of respiratory excursion where the motion platform moves in up (in)–down (out) direction.

**FIG. 3:**
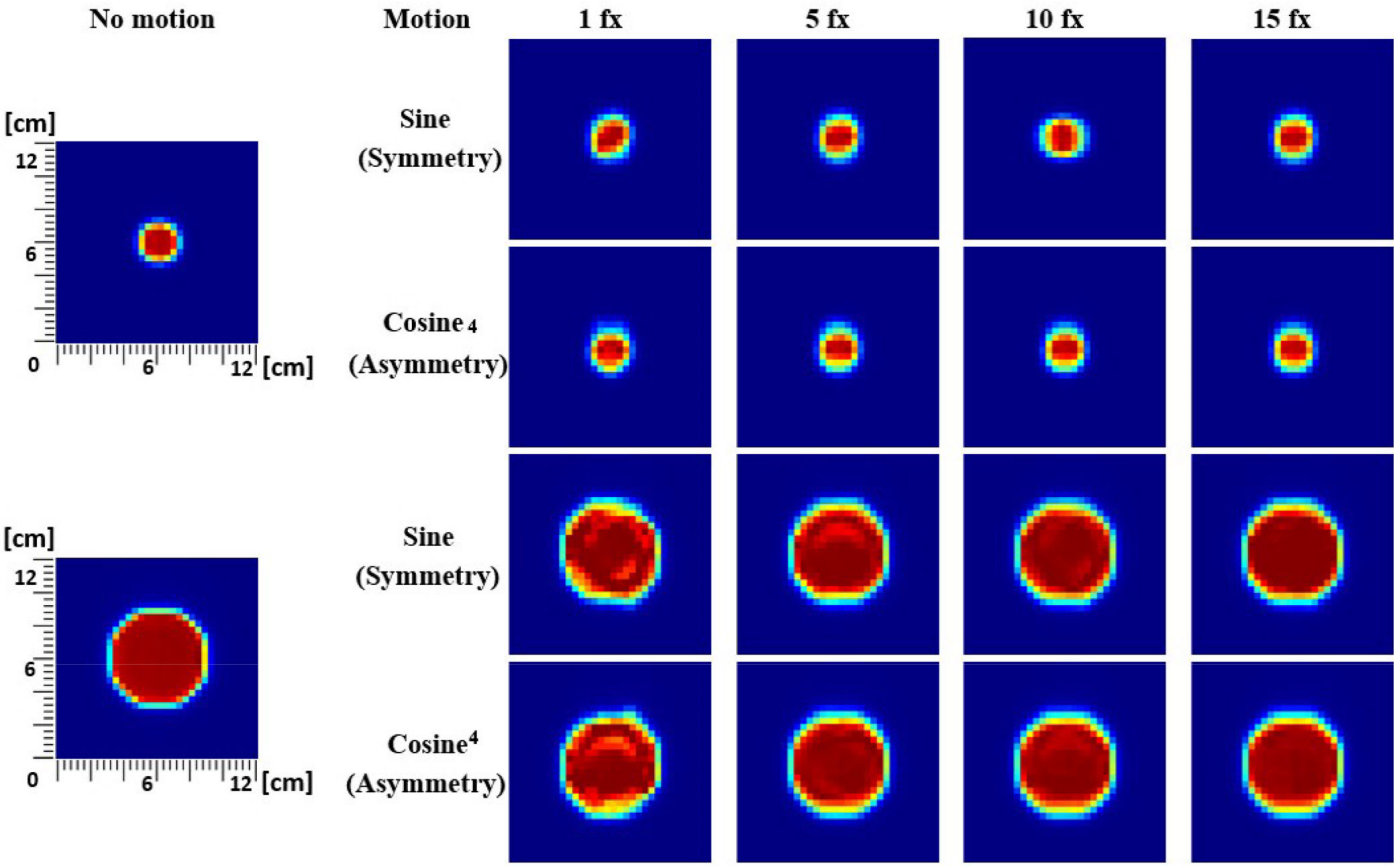
Two‐dimensional array ion chamber measurements of dose distribution with 1 cm motion amplitude (2 cm respiratory excursion) with a fixed 5‐second breathing cycle for targets of 3 cm and 10 cm diameter spheres at 5 cm depth for sinusoidal symmetric (sine) and realistic asymmetric (cosine^4^) breathing patterns as a function of fractions. The motion platform moves in up (in)–down (out) direction here.

In order to estimate the degradation of target dose coverage due to respiratory motions, we evaluated dose conformity and uniformity of each measurement dataset at the center plane of each size of moving targets simulated by the motion platform. Figure 2 reveals qualitative deterioration of dose delivery at the target in the plane of the simulated motion. Larger motions show more severe loss of conformity as well as inhomogeneity of the dose distribution on the target. Both conformity and homogeneity, however, were significantly recovered as fractionation increased as shown in Fig. 3. To further quantify two different simulated breathing motions, interplay effects of various parameters, and how fractionation mitigates the degradation of dose conformity and uniformity differently for those breathing patterns, CN and HI metrics were used in following subsections.

### 3.A Impact of motion on dose conformity

**FIG. 4:**
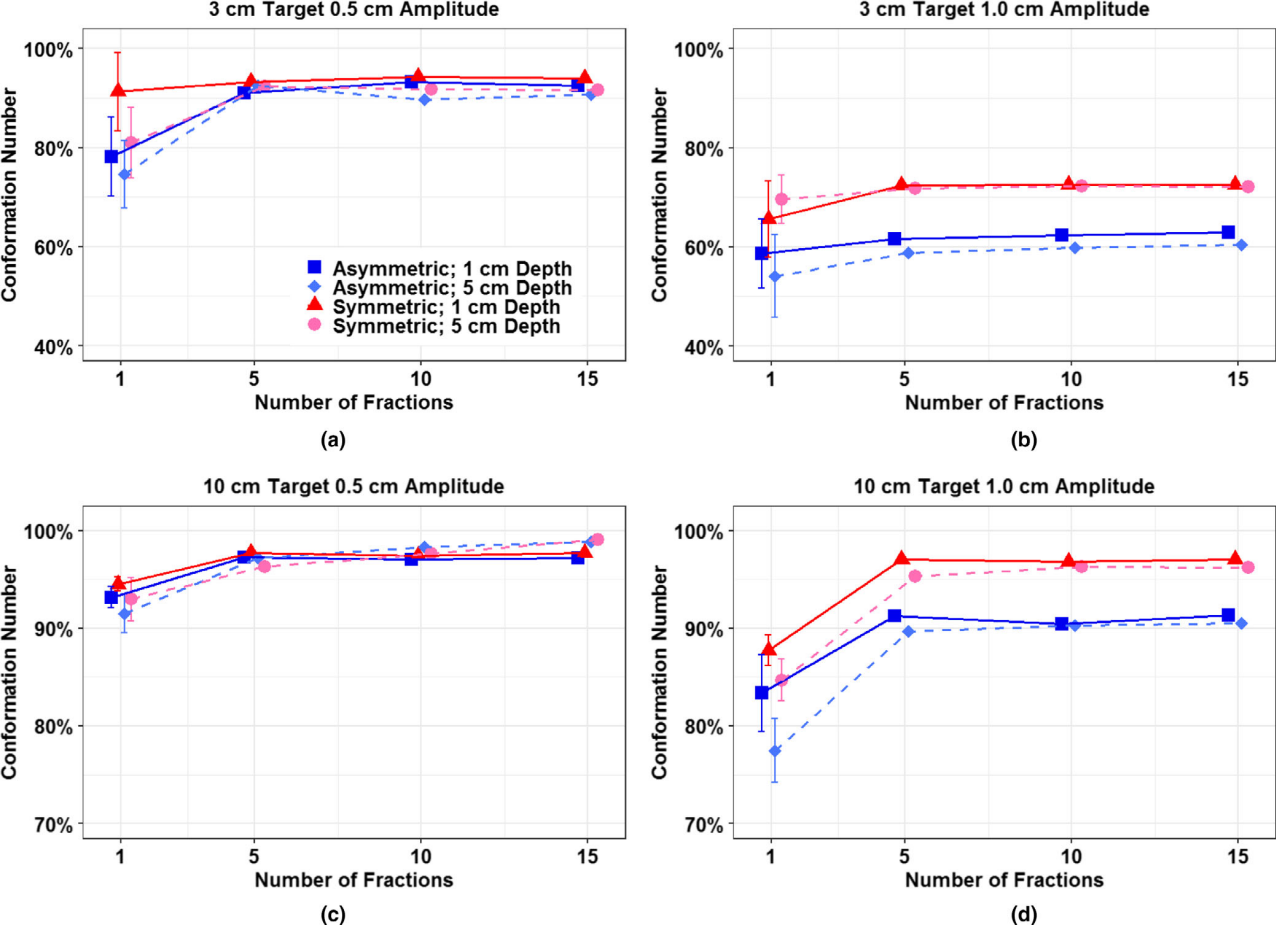
Relative conformation numbers normalized to the values with no motion for all measurement dataset as a function of fraction. For a single fraction measurement point is represented with the mean value of 15 independent measurements with error bars (±σ).

Absolute CNs for all dataset are listed in Table 1 including CNs with no motion. Figure 4 shows relative CNs in percentage, which were normalized to the values without motion to show how much conformity index values are affected by simulated motions. Overall conformity numbers improve as the number of fractions increases regardless of breathing patterns, target sizes, or target locations in terms of depths.

**TABLE 1:** Absolute conformation numbers for all dataset.

Target Diameter (cm)	Depth (cm)	Breathing Pattern	Amplitude (cm)	1 fx	5 fx	10 fx	15 fx	No Motion
3	5	Cosine^4^ (Asymmetric)	0.5	0.68 ± 0.06	0.84	0.82	0.83	0.91
			1.0	0.49 ± 0.08	0.54	0.54	0.55	
		Sine (Symmetric)	0.5	0.74 ± 0.06	0.84	0.84	0.84	
			1.0	0.64 ± 0.04	0.66	0.66	0.66	
	1	Cosine^4^ (Asymmetric)	0.5	0.72 ± 0.07	0.84	0.86	0.86	0.93
			1.0	0.54 ± 0.06	0.57	0.58	0.58	
		Sine (Symmetric)	0.5	0.85 ± 0.07	0.90	0.90	0.90	
			1.0	0.61 ± 0.07	0.65	0.65	0.65	
10	5	Cosine^4^ (Asymmetric)	0.5	0.87 ± 0.02	0.92	0.94	0.94	0.95
			1.0	0.74 ± 0.03	0.85	0.86	0.86	
		Sine (Symmetric)	0.5	0.88 ± 0.02	0.92	0.93	0.94	
			1.0	0.81 ± 0.02	0.91	0.92	0.92	
	1	Cosine^4^ (Asymmetric)	0.5	0.89 ± 0.01	0.93	0.93	0.93	0.96
			1.0	0.80 ± 0.04	0.87	0.86	0.87	
		Sine (Symmetric)	0.5	0.91 ± 0.01	0.94	0.94	0.94	
			1.0	0.84 ± 0.02	0.92	0.92	0.92	

5 cm WET range shifter used for targets at 1 cm depth. Breathing cycle fixed at 5 sec. Measurements with no motion for reference. For 1 fraction, mean and standard deviation from 15 independent measurements listed

For small targets with small amplitude as shown in [Fig. 4(a)], conformity values represented by relative CNs reached over 90% of those without motion. For small targets with relatively large amplitude as in [Fig. 4(b)], target is significantly under‐dose with below 70% of the conformity of non‐moving target doses for a single fraction. Adding fractions has a certain limit on mitigating target dose conformity loss. Large targets as in [Figs. 4(c) and 4(d)] showed similar trends to the small targets, but with higher conformity numbers. Large targets with amplitudes up to 1 cm achieved over 95% of the conformity of stationary targets as fractions increased except the case of asymmetric breathing with large amplitude (2 cm respiratory excursion), which clearly deteriorated the dose distribution with less recovery (90%) as a function of fraction. These distinctive characteristics of conformity value recovery with fractions between breathing patterns were also observed in small targets with large motion as shown in [Fig. 4(b)]. Realistic asymmetric breathing patterns achieved less conformity as the number of fractions increased than sinusoidal symmetric motion. Note that spot sizes according to different target depths had negligible difference on interplay effect on the target dose conformity.

### 3.B Impact of motion on dose homogeneity

**FIG. 5:**
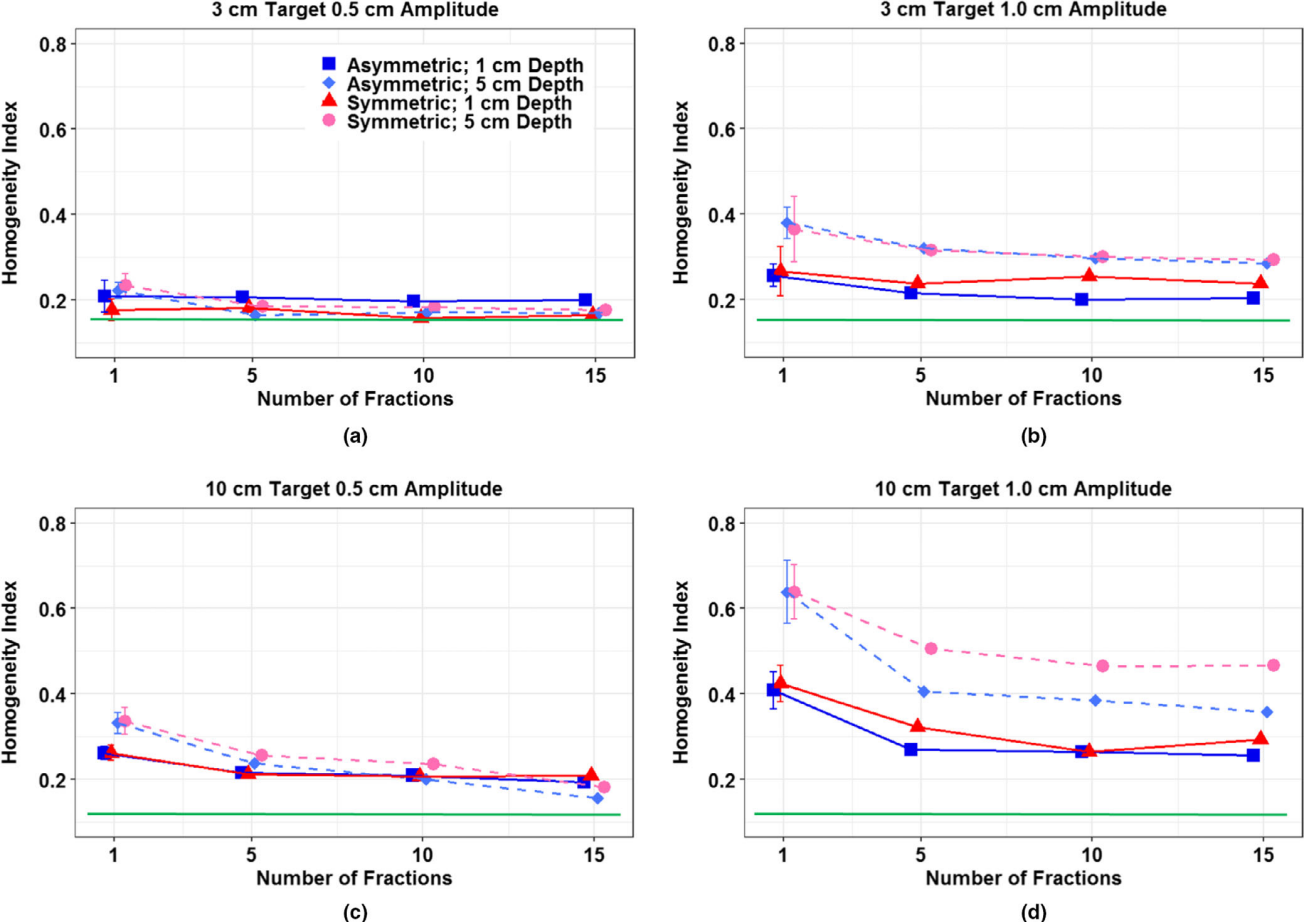
Homogeneity index for all measurement dataset as a function of fraction. For a single fraction measurement point is represented with the mean value of 15 independent measurements with error bars (±σ). Note that green line represents HI values for static targets.

HI values were shown in Table 2. Figure 5 shows homogeneity as an estimate of the dosimetric influence due to the patterns of respiratory motion as a function of fraction where green lines were added for each plot to represent HI values for static targets as a reference. Overall homogeneity improved as more fractions were added. For small amplitude, there are only slight differences in the achieved homogeneities regardless of target size, location, breathing pattern, or amplitude as shown in [Figs. 5(a) and 5(c)]. For large target, both small and large motion amplitude showed the similar trend. Interplay effects due to respiratory motion were more affected by spot sizes than breathing patterns. In [Figs. 5(b) and 5(d)], larger spots on the target at shallow location show better homogeneity than smaller spot sizes in the target at deeper locations.

**TABLE 2:** Homogeneity index for all dataset.

Target Diameter (cm)	Depth (cm)	Breathing Pattern	Amplitude (cm)	1 fx	5 fx	10 fx	15 fx	No Motion
3	5	Cosine^4^ (Asymmetric)	0.5	0.22 ± 0.02	0.16	0.17	0.17	0.16
			1.0	0.38 ± 0.04	0.32	0.30	0.28	
		Sine (Symmetric)	0.5	0.23 ± 0.03	0.18	0.18	0.18	
			1.0	0.36 ± 0.08	0.32	0.30	0.29	
	1	Cosine^4^ (Asymmetric)	0.5	0.21 ± 0.04	0.21	0.20	0.19	0.16
			1.0	0.26 ± 0.03	0.22	0.20	0.20	
		Sine (Symmetric)	0.5	0.18 ± 0.03	0.22	0.20	0.20	
			1.0	0.27 ± 0.06	0.24	0.26	0.24	
10	5	Cosine^4^ (Asymmetric)	0.5	0.33 ± 0.02	0.24	0.20	0.16	0.12
			1.0	0.64 ± 0.07	0.40	0.38	0.36	
		Sine (Symmetric)	0.5	0.34 ± 0.03	0.26	0.24	0.18	
			1.0	0.64 ± 0.06	0.51	0.46	0.47	
	1	Cosine^4^ (Asymmetric)	0.5	0.26 ± 0.01	0.22	0.21	0.19	0.12
			1.0	0.41 ± 0.04	0.27	0.26	0.26	
		Sine (Symmetric)	0.5	0.26 ± 0.02	0.21	0.21	0.21	
			1.0	0.42 ± 0.04	0.32	0.26	0.26	

5 cm WET range shifter used for targets at 1 cm depth. Breathing cycle fixed at 5 sec. Measurements with no motion for reference. For 1 fraction, mean and standard deviation from 15 independent measurements listed.

## 4 Discussion

A main factor impacting dose delivery accuracy for the moving targets is the interplay effect including the irregularity of respiratory motion during PBS delivery in proton therapy treatment. As shown in several studies,^1^, ^2^, ^3^ the interplay effect is normally expected to decrease as the fraction number increases. In this study, for most cases, improvement in dose deterioration due to simulated respiratory motions was also observed as the fraction number increased, which were quantified in terms of conformity as well as homogeneity indices. The exception was observed in the case of relatively large motion such as in the patient with deep breathing on the small target, where there was substantial dose deterioration, and so increasing fractions were unable to improve the dose conformity or homogeneity. In this case, additional motion management strategies, such as breath‐hold or gating, may be warranted. This finding confirmed other simulation study result^2^ by measurement‐based experiments.

Two main parameters — breathing patterns and spot sizes — and their impact on target dose distributions were studied. Breathing patterns were represented by sinusoidal symmetric and more realistic asymmetric motions. Spot sizes were varied due to both the different target locations in depth and the use of range shifters. Breathing patterns had more impact on the conformity, but less impact on the homogeneity of dose distribution as shown in [Figs. 4(b) and 4(d)]. This is because spending more time in a certain breathing phase such as non‐sinusoidal asymmetric (64% on expiration in our motion simulation) causes geometrical offset or miss of dose delivery to the target. In this study, we did not control the initial phase of the motion platform since variations with initial phases had rather random impact on interplay effects whereas the motion amplitude dominated the general trend of interplay effects.^1^

In comparison, dose homogeneity was more impacted by intrinsic beam spot characteristics. Dose heterogeneity was more pronounced with smaller spots under the same breathing patterns as shown in [Figs 5(b) and 5(d)]. For shallow targets, range shifters had to be used to reduce the range of the lowest energy proton beams (70 MeV corresponding to the range of ~ 4.1 cm). These significantly broaden spot size leading to fewer spots with bigger sizes and larger penumbras, which is less sensitive to motion, achieving better homogeneity whereas surrounding organs may be at higher risk of overdose. This result is also consistent with a Monte Carlo study by Grassberger et al*.* 28 For relatively small motion amplitudes, the magnitude of interplay effects in terms of dose homogeneity can be effectively decreased by increasing pencil beam widths and therefore overlap between different scanning positions.

We found that fractionation alone has some limitation in mitigating the interplay effect in terms of dose conformity, especially in non‐sinusoidal asymmetric motion compared to symmetric motion. In addition, Bert et al^29^ showed that the most dominant parameter influencing interplay patterns is the motion amplitude and changes in motion parameters or scanning parameters resulted in almost unpredictable dose heterogeneity. Therefore, a well‐defined selection of adequate margins and motion management are required to minimize the interplay effect of PBS treatment.

There are few drawbacks of our experimental setup. The study design was to investigate PBS interplay effect with a simple geometric shape of moving target in a homogeneous water phantom and to evaluate the motion‐affected dose distributions in 2D plane measurements. This could only represent a surrogate to quantify the volumetric plan quality. Moreover, the interplay effect of PBS delivery with irregular target geometry under realistic patient‐specific breathing motion in high degree of heterogeneity of real patient body may be much more complicated to quantify.

## 5 Conclusions

In this study, the dosimetric interplay effects of different breathing patterns and spot sizes were investigated based on physical measurements. More fractions generally helped mitigate the degradation of dose conformity as well as homogeneity due to respiratory motions. Our study has also confirmed that it is possible to treat moderately moving targets with motion amplitude less than 5 mm using pencil beam scanning in standard fractionation. For the case of small target under relatively large motions, especially with irregular breathing patterns or asymmetric motions where relatively significant amount of time is spent on a certain breathing phase such as end exhale, care must be taken such as further study on breathing patterns for each patient as well as adequate use of motion management.

## Conflict of Interest

The authors report no conflicts of interest.
